# Insights Into Bifenthrin Stereoisomers and Their Regulatory Implications

**DOI:** 10.1002/open.70212

**Published:** 2026-04-20

**Authors:** Nayara C. M. Santos, Nayara D. Coutinho, Vitor S. Duarte, Anderson Catao, Antônio S. N. Aguiar, Lucas D. Dias, James O. Fajemiroye, Hamilton B. Napolitano

**Affiliations:** ^1^ Laboratório de Polimorfismo Molecular Faculdade SENAI Roberto Mange Anápolis GO Brazil; ^2^ Theoretical and Structural Chemistry Group State University of Goiás Anápolis GO Brazil; ^3^ Laboratório de Novos Materiais Universidade Evangélica de Goiás Anápolis GO Brazil; ^4^ Institute of Biological Sciences Federal University of Goiás Goiânia GO Brazil

**Keywords:** bifenthrin, density functional theory, Hirshfeld surface, regulatory framework

## Abstract

Bifenthrin (BF) is a synthetic pyrethroid insecticide extensively applied in agricultural and urban pest control. The molecular structure contains two *stereogenic* centers, giving rise to four stereoisomers with distinct physicochemical, electronic, and biological properties. However, many regulatory frameworks still consider BF as a single racemic active substance. Herein, we present a comprehensive structural and theoretical investigation of BF, integrating solid‐state analysis with electronic structure calculations based on density functional theory. Also, supramolecular arrangements were analyzed using Hirshfeld surface, quantum theory of atoms in molecules, and natural bond orbital approaches. In parallel, global and local reactivity descriptors were evaluated through frontier molecular orbital analysis, molecular electrostatic potential mapping, and Fukui indices. Finally, the findings reveal stereoelectronic differences among the BF stereoisomers, with the 1*S*,3*S* isomer displaying a markedly anisotropic charge distribution and enhanced electronic reactivity, thereby underscoring the importance of incorporating stereochemical knowledge into pesticide risk assessment.

## Graphical abstract

Bifenthrin is a pyrethroid insecticide with two stereogenic centers, generating four stereoisomers with distinct physicochemical and electronic properties. A combined crystallographic and density functional theory study, supported by Hirshfeld surface, quantum theory of atoms in molecules, natural bond orbital, and reactivity descriptor analyses, reveals stereoelectronic differences among the isomers, highlighting the enhanced electronic reactivity of the 1*S*,3*S* form and its implications for pesticide risk assessment.

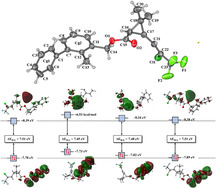

## 1 Introduction

Bifenthrin (BF), a synthetic third‐generation pyrethroid, has been widely utilized in agricultural and urban pest control since the early 1980s, largely due to its rapid knockdown effect, contact and stomach toxicity, and prolonged residual activity. Its increasing adoption has been driven by regulatory restrictions on organophosphate insecticides and its favorable properties, including lower dermal irritation and enhanced chemical stability among other pyrethroids [1, 2, 3]. These characteristics have positioned BF as a key active ingredient in various commercial formulations for integrated pest management [2, 4]. Chemically, BF exhibits a complex molecular structure composed of substituted aromatic rings, a vinyl group, and an ester linkage, with a molecular formula of C_23_H_22_ClF_3_O_2_ [5]. The molecule contains two stereogenic centers, giving rise to four stereoisomers (two pairs of enantiomers) with distinct biological activities. Enantioselective toxicity has been reported in multiple studies, indicating that certain enantiomers may exert greater cytotoxic or endocrine‐disrupting effects [6, 7, 8, 9]. Its physicochemical behavior, including solubility, crystal packing, and environmental persistence, is strongly influenced by its stereochemistry and intermolecular interactions. Advanced computational methods, such as density functional theory (DFT) [10, 11], along with crystallographic techniques like Hirshfeld surface (HS) analysis, have been employed to investigate these properties and support the rational design of BF‐based formulations [5, 12, 13, 14].

The regulatory landscape for BF varies significantly across jurisdictions, reflecting distinct environmental priorities and risk assessment methodologies. In the United States, BF is regulated under the Federal Insecticide, Fungicide, and Rodenticide Act (FIFRA) and is subject to periodic review by the Environmental Protection Agency (EPA), which has implemented mitigation measures to reduce ecological risks, particularly to pollinators and aquatic organisms [2]. In Brazil, BF is regulated by the Brazilian Institute of Environment and Renewable Natural Resources (IBAMA) under Law No. 7.802/89 and Decree No. 4.074/02, with its first product approval dating back to 1998 [15, 16, 17, 18]. Similar to other jurisdictions, the regulatory framework does not differentiate between its stereoisomeric forms. In contrast, the European Union withdrew approval of BF due to its high aquatic toxicity and bioaccumulation potential, as formalized in Commission Delegated Regulation (EU) 2022/643 [19]. Despite these actions, regulatory frameworks in both regions treat BF as a single active substance, without accounting for its stereoisomeric complexity. Scientific evidence demonstrates that BF enantiomers differ in toxicity, environmental persistence, and molecular reactivity [1, 4, 6]. However, current regulations do not require stereospecific data or polymorph characterization. This gap is particularly relevant given that commercial formulations consist of racemic mixtures, which may amplify adverse effects.

**FIGURE 1:**
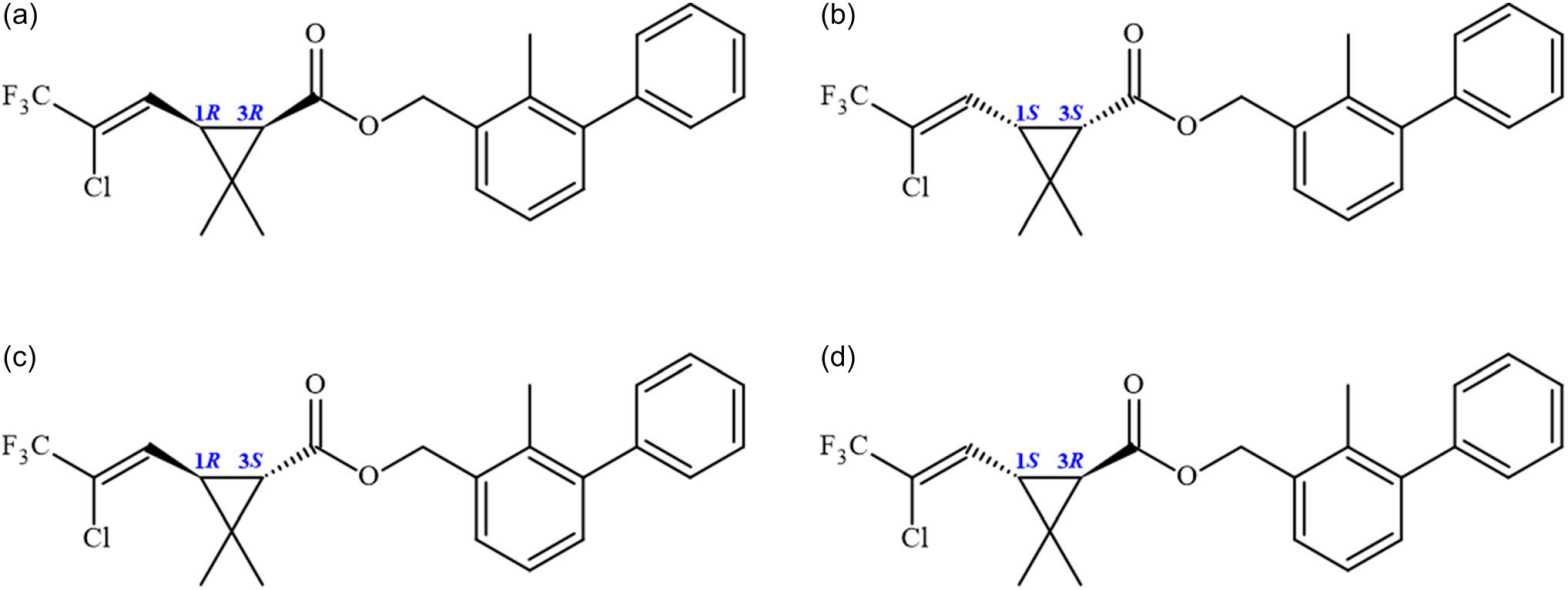
2D molecular structures of BF highlighting the configurations at the two stereogenic centers (C_1_ and C_3_): (a) 1*R*,3*R*; (b) 1*S*,3*S*; (c) 1*R*,3*S*; and (d) 1*S*,3*R*.

The absence of stereochemical considerations in pesticide regulation underscores the need for updated policies that integrate molecular‐level insights into risk assessment, promoting safer and more sustainable agrochemical practices [5, 7, 8, 9]. However, only 1*R*,3*R* and 1*S*,3*S* have been extensively studied (Figure 1) [1, 7, 9, 20]. The isomers of BF exhibit distinct biological activities and toxicological profiles, with the compound displaying *cis* *–trans* isomerism. The stereochemical complexity of BF significantly influences its environmental behavior, insecticidal efficacy, and safety profile. This structural complexity warrants in‐depth *molecular‐level* investigations to better understand its physicochemical properties and toxicological implications [3, 7]. Chirality is a key factor in the efficacy and safety of insecticides, as enantiomers can exhibit marked differences in toxicity, selectivity, and environmental behavior [21, 22]. Studies have shown that these variations influence both insecticidal activity and risks to nontarget organisms [21, 22, 23, 24]. Stereospecific analysis enables the identification of safer and more effective isomers, providing a foundation for sustainable formulations and regulatory policies based on molecular‐level evidence [7, 23, 24].

BF is a chiral pyrethroid insecticide commercially formulated as a racemic mixture of the *cis* isomers 1*R*,*3R* and 1*S*,*3S*. Increasing scientific evidence has demonstrated that these enantiomers exhibit distinct biological activities and toxicological profiles, raising concerns about their environmental and human health impact. Liu et al. [1], demonstrated that 1*S*,*3S* is significantly more toxic to human cells, inducing apoptosis, DNA damage, oxidative stress, and cytoskeletal disruption, whereas 1*R*,*3R* showed minimal effects [7]. This author also reported that 1*S*,*3S* selectively interferes with hormone synthesis in rat ovarian granulosa cells, reducing progesterone and prostaglandin E2 (PGE2) production via inhibition of the protein kinase C (PKC) signaling pathway [20]. In contrast, studies on aquatic organisms revealed that 1*R*,*3R* is up to 300 times more toxic to the target insect *Pieris rapae* and significantly more active against *Ceriodaphnia dubia*, highlighting a reversed enantioselectivity between target and nontarget species [1]. Liao et al. [6] demonstrated that 1*S*,*3S* induces more extensive metabolic disturbances in human hepatocytes, affecting pathways related to amino acid metabolism, energy production, and nucleotide synthesis [6]. Furthermore, 1*S*,*3S* undergoes preferential microbial degradation in sediments, resulting in the environmental enrichment of the more toxic 1*R*,*3R* [1]. These findings underscore the importance of investigating BF at the molecular and structural level, considering its stereochemistry as a critical factor influencing efficacy, toxicity, and environmental behavior [1, 6, 7, 20, 24]. Enantiomeric variation can affect insecticidal activity, with less stable forms sometimes showing greater potency. Computational methods, such as DFT and thermodynamic modeling, are effective tools for analyzing molecular properties and improving crystallization processes [14, 25, 26].

This study aims to provide a comprehensive structural and theoretical characterization of the BF molecule through solid‐state analysis and DFT‐based electronic structure calculations. Frontier molecular orbitals (FMO) and molecular electrostatic potential (MEP) maps were undertaken, and reactive sites were identified using the Fukui function [27, 28]. In addition, solid‐state investigations using HS analysis and quantum theory of atoms in molecules (QTAIM) were employed to elucidate intermolecular interactions [29, 30]. This integrated approach aims to provide a microscopic structural and electronic interpretation of BF stereoisomers, establishing correlations between their stereoelectronic features and physicochemical properties. These insights may help rationalize differences reported in the literature and provide additional perspective on regulatory considerations associated with chiral pesticides.

## 2 Computational Procedures

### 2.1 Solid‐State Description

The solid‐state description allows for the understanding of intermolecular interaction patterns within the supramolecular arrangement, enabling the acquisition of relevant information regarding the stability of the substance, its physicochemical properties, and functional performance. The crystal structure of BF was obtained from the Cambridge Structural Data Centre (CCDC) under the deposition code 2142944 [9]. It corresponds to a racemic mixture in which the enantiomers 1*R*,3*R* and 1*S*,3*S* are present. To date, the solid‐state description of the 1*R*,3*S* and 1*S*,3*R* racemates has not been reported in the literature. For the solid‐state study of BF, the Mercury program [31] was employed for molecular structure visualization and analysis of intermolecular interactions; CrystalExplorer [32] was used for the determination of HS and the construction of 2D fingerprint plots; and ORTEP [33] was applied for the visualization of anisotropic displacement ellipsoids. Figure 1 presents the 2D representations of the BF stereoisomers.

HS analysis enables the qualitative and quantitative visualization of intermolecular contacts in the solid‐state based on atomic electron densities [32, 34]. The HS is defined through the Hirshfeld weight function, W(r), which represents the fractional contribution of the electron density of a reference molecule relative to the total electron density of the crystal at a given point r. The normalized contact distance, $d_{\text{norm}}$, is employed to identify regions of significant intermolecular interaction on the HS [32, 34, 35]. Additionally, 2D fingerprint plots were generated to identification and quantitative assessment of specific intermolecular contact types, such as H···H, H···F, and C···H interactions [35, 36]. Additionally, ORTEP [37, 38] was employed to generate crystallographic representations of the BF molecule, allowing the visualization of its molecular geometry together with anisotropic displacement ellipsoids. These ellipsoids provide information on the magnitude and direction of atomic thermal motion within the crystal lattice, thereby offering insights into structural reliability, possible disorder, and conformational features in the solid state. The ORTEP representations were used exclusively for structural illustration and qualitative assessment of the refined crystallographic model [33].

### 2.2 Molecular Modeling

The molecular structures of the four BF stereoisomers shown in Figure 1 were investigated to obtain insights into their chemical reactivity. The atomic coordinates of the 1*R*,3*R* and 1*S*,3*S* isomers were obtained directly from the solid‐state structure, whereas the remaining stereoisomers were constructed using the GaussView 6.0 program [39]. All molecular geometries were optimized using DFT [40, 41] as implemented in the Gaussian 16 software package [42]. Geometry optimizations were carried out at the M06‐2X/6‐311++G(d,p) level of theory. Previous studies have demonstrated that the M06‐2X [43] functional provides an accurate description of medium‐range electron correlation and noncovalent interactions, making it a reliable choice for modeling thermodynamic and reactivity‐related properties of chemical systems [44]. Frequency calculations were subsequently performed to confirm that all optimized structures corresponded to true ground‐state *minima*, characterized by the absence of imaginary frequencies. After full geometry optimization, the energies of the FMOs, namely the highest occupied molecular orbital (HOMO) and the lowest unoccupied molecular orbital (LUMO), were obtained. Based on these orbital energies, several global chemical reactivity descriptors were calculated, including the energy gap ($\Delta E_{H-L}$), which is associated with the kinetic stability of the molecule; the chemical hardness [45, 46],

(1) $$\eta =\frac{1}{2}{\left(\frac{\partial^{2}E}{\partial N^{2}}\right)}_{\upsilon (r)}\approx \frac{I-A}{2}$$

which reflects the resistance of the electron cloud to deformation during chemical processes; the chemical potential [46],

(2) $$\mu ={\left(\frac{\partial E}{\partial N}\right)}_{\upsilon (r)}\approx -\frac{I+A}{2}=-\chi$$

related to the tendency for charge transfer between species; and the global electrophilicity index [47],

(3) $$\omega =\frac{\mu^{2}}{2\eta }$$

which measures the stabilization energy of a system upon acquiring electronic charge from its environment. In Equations (1) and (2), E is the energy of the system, N is the number of particles, υ is the external potential, χ is the electronegativity, $I\approx -E_{\text{HOMO}}$ is the ionization potential, and $A\approx -E_{\text{LUMO}}$ is the electron affinity.

MEP surfaces [48, 49], along with the corresponding electrostatic potential, V(r), values,



(4)

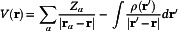




were obtained to identify the local nucleophilic and electrophilic regions of each stereoisomer. In Equation (4), $Z_{\alpha }$ denotes the charge of nucleus α located at position $r_{\alpha }$, and ρ(r′) represents the electronic charge density at position r′ [48]. The MEP surfaces were generated using the GaussView 6.0 program, whereas the V(r) values were obtained using the Multiwfn 3.8 software [50]. Furthermore, through the Fukui function [51, 52], defined by Equation (5),

(5) $$f\left(r\right)={\left[\frac{\partial \rho \left(r\right)}{\partial N}\right]}_{\upsilon (r)}$$

was employed to predict local reactivity sites, allowing the determination of indices associated with nucleophilic, electrophilic, and radical attacks. The Fukui function describes the local sensitivity of the electron density at a point r in space in response to variations in the number of electrons in the system, thereby indicating regions that are more prone to nucleophilic, electrophilic, or radical attack processes. In Equation (5), ρ(r) represents the electron density at position r, N denotes the total number of electrons in the system, and v corresponds to the external potential, which is kept constant during the differentiation.

All intermolecular interactions were analyzed using topological parameters derived from QTAIM [53, 54]. To characterize the nature of these interactions, single‐point calculations were performed using atomic coordinates directly extracted from the experimental crystal structure, without further geometry optimization, to preserve the solid‐state environment. All calculations were carried out at the same level of theory employed for the electronic‐structure analyses. The topological properties were evaluated using the Multiwfn 3.8 program [50]. In addition, the stability of the intermolecular interactions was further examined through natural bond orbital (NBO) analysis [55, 56]. Donor–acceptor interactions were quantified by estimating the stabilization energies associated with charge transfer using second‐order perturbation theory [57],

(6) $$E_{i\to j^{*}}^{\left(2\right)}=-n_{\sigma }\frac{\left\langle \sigma_{i}|\hat{F}|\sigma_{j^{*}}\right\rangle }{\varepsilon_{j^{*}}-\varepsilon_{i}}=-n_{\sigma }\frac{F_{ij}^{2}}{\varepsilon_{j^{*}}-\varepsilon_{i}}$$

where $\left\langle \sigma_{i}|\hat{F}|\sigma_{j^{*}}\right\rangle$ (or $F_{ij}^{2}$) is the Fock matrix element between the i and j NBOs, $\varepsilon_{\sigma^{*}}$ is the energy of the antibonding orbital $\sigma^{*}$, and $\varepsilon_{\sigma }$ is the energy of the bonding orbital $\sigma ;n_{\sigma }$ is the population occupation of the σ donor orbital.

## 3 Results and Discussion

### 3.1 Solid‐State Description

BF contains two chiral carbons (two stereogenic centers) in its structure, which gives rise to four possible stereoisomers: (1*R*,3*R*), (1*S*,3*S*), (1*R*,3*S*), and (1*S*,3*R*). The first two correspond to the *cis* configuration, whereas the latter two correspond to the *trans* configuration. However, in the crystalline state, only racemic pairs are observed, occurring either as mixtures of (1*R*,3*R*)/(1*S*,3*S*) or (1*R*,3*S*)/(1*S*,3*R*) enantiomers [9, 58]. Commercially, BF is predominantly marketed in the technical *cis* form, which contains ≈97% of the *cis* isomer and only 3% of the *trans* isomer. Consequently, this formulation represents a racemic mixture of the enantiomers (1*R*,3*R*) and (1*S*,3*S*) [58]. The solid‐state structure of BF reported in the literature (CCDC 2 142 944) corresponds to a racemate composed of the (1*R*,3*R*) and (1*S*,3*S*) enantiomers in an ≈50:50 ratio [9]. This structure crystallizes in the monoclinic crystal system, space group C2/c, with eight molecules per unit cell (Z = 8).

**FIGURE 2:**
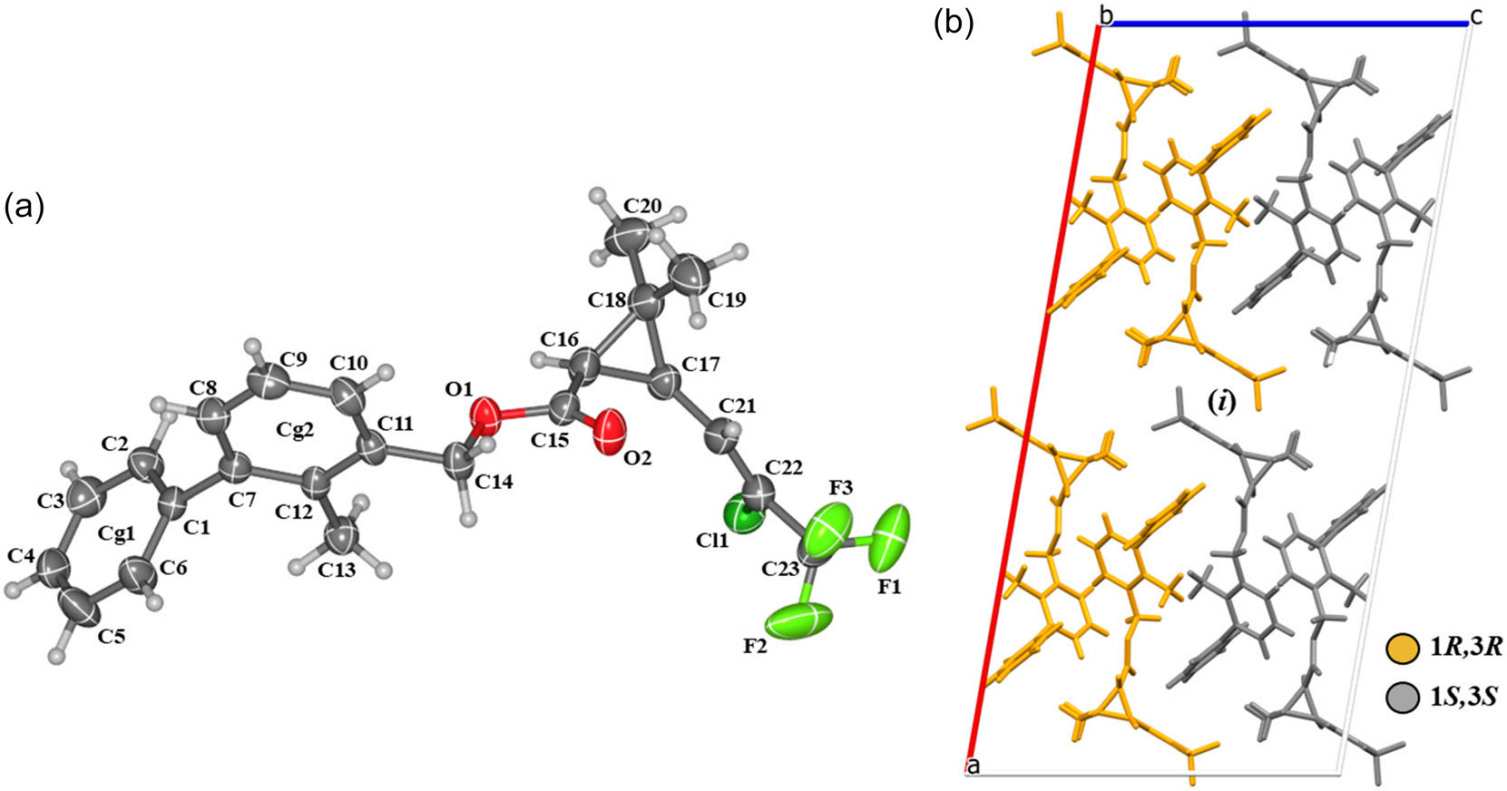
(a) ORTEP representation of BF with thermal ellipsoids at 50% probability; H atoms are included and shown as spheres of arbitrary radius. (b) Molecular packing in the unit cell (along the *b‐*axis) showing the 1*R*,3*R* and 1*S*,3*S* enantiomers. (*i*) inversion center. Hydrogen atoms are depicted as spheres of arbitrary radius for clarity.

Because BF crystallizes in a centrosymmetric space group, the presence of an inversion center within the unit cell necessarily requires the coexistence of both enantiomers (1*R*,3*R* and 1*S*,3*S*) in the crystal lattice. The inversion operation transforms each molecule into its mirror image, ensuring that the individual molecular chirality is retained while the crystal becomes racemic (Figure 2).

**FIGURE 3:**
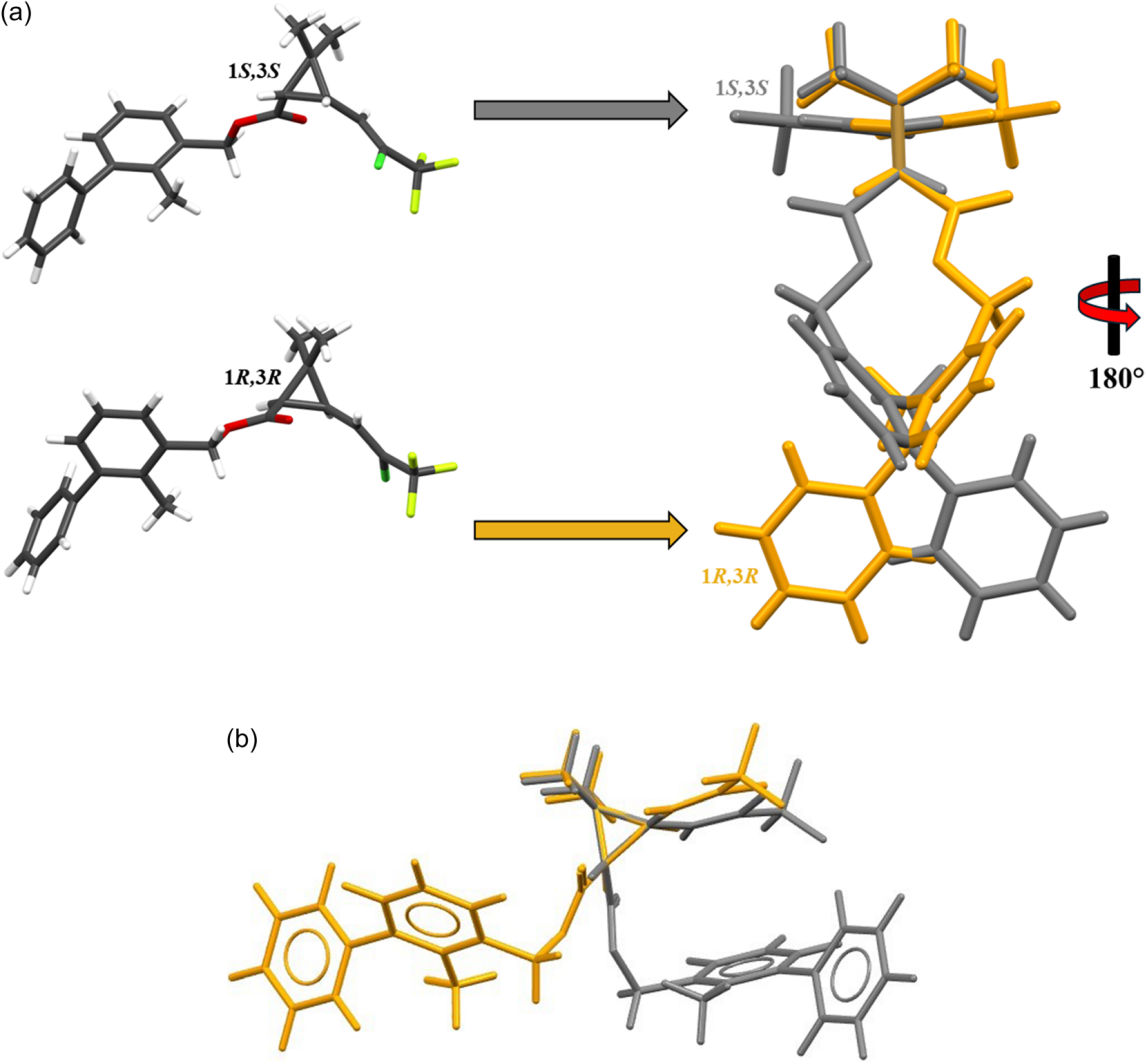
(a) Superposition of the 1*R*,3*R* and 1*S*,3*S* enantiomers of BF in the crystalline state (RMSD = 3.22 × 10^–15^) and (b) ground‐state optimized structures obtained at the M06‐2X level of theory (RMSD = 0.00927). The 1*R*,3*R* and 1*S*,3*S* stereoisomers are represented by gold and gray, respectively.

The BF stereoisomers in the crystalline state exhibit nonplanar molecular structures. The overlay of the (1*R*,3*R*) and (1*S*,3*S*) enantiomers reveals that the dihedral angles of the stereogenic centers have equal magnitudes but opposite signs ($\theta_{RR}=-\theta_{SS}$), differing by ≈180°, as expected for an enantiomers pair (Table 1). This behavior confirms their mirror‐image relationship, the opposite chiral configurations at the stereogenic centers, and the lack of superimposability between the enantiomers, in agreement with the centrosymmetric nature of the crystal (Figure 3a). In general, enantiomers share identical physical properties, such as molecular weight, bond lengths, and bond angles. However, their opposite chiral configurations may lead to differences in certain physicochemical behaviors, particularly in interactions with chiral reagents or biological systems [58, 59, 60]. Since BF occurs as a racemate, chirality‐dependent interactions are effectively averaged, whereas properties that do not depend on chirality, such as density, melting point, and solubility in achiral solvents, remain essentially unchanged [61, 62]. Nevertheless, racemic crystals may exhibit subtle differences when compared to the pure enantiomers.

**TABLE 1:** Dihedral angles (*θ*) for 1*R*,3*R* and 1*S*,3*S* enantiomers of BF.

**Dihedral angles (°)**	**Experimental**		**Theoretical**		
	**1*R*,3*R***	**1*S*,3*S***	**1*R*,3*R***	**1*S*,3*S***	
C_22_–C_21_–C*_17_–C*_16_	131.72	−131.72	153.55	−146.74	
C_12_–C_7_–C_1_–C_6_	−32.34	−90.76	56.72	56.85	
C_12_–C_11_–C_14_–O_1_	75.51	111.25	74,59	69,41	
C_11_–C_14_–O_1_–C_15_	164.59	28.40	79,75	69,09	
O_1_–C_15_–C*_16_–C*_17_	−151.17	151.17	−153.06	116.39	
C*_17_–C_21_–C_22_–C_23_	178.64	−178.64	179.44	−174.15	

* Stereogenic centers.

Moreover, because BF contains two stereogenic centers, the properties of the 1*R*,3*R*/1*S*,3*S* racemate may differ from those of the 1*R*,3*S*/1*S*,3*R* racemate [63, 64]. The BF molecule contains seven rotatable bonds, and relaxed conformational scan calculations indicated that both the 1*R*,3*R* and 1*S*,3*S* enantiomers do not exhibit inverted conformations in their respective ground states. Subtle rotations were observed around the C_7_—C_1_, C_11_—C_14_, and C_14_—O_1_ bonds. In contrast, more pronounced rotations occur around the C_15_—C_16_, C_17_—C_21_, and C_21_—C_22_ bonds, leading to a more twisted conformation of the 1S,3S molecule. This conformational distortion favors the formation of intramolecular interactions between the aromatic region and the CCl–CF_3_ moiety, as shown in Figure 3b.

**FIGURE 4:**
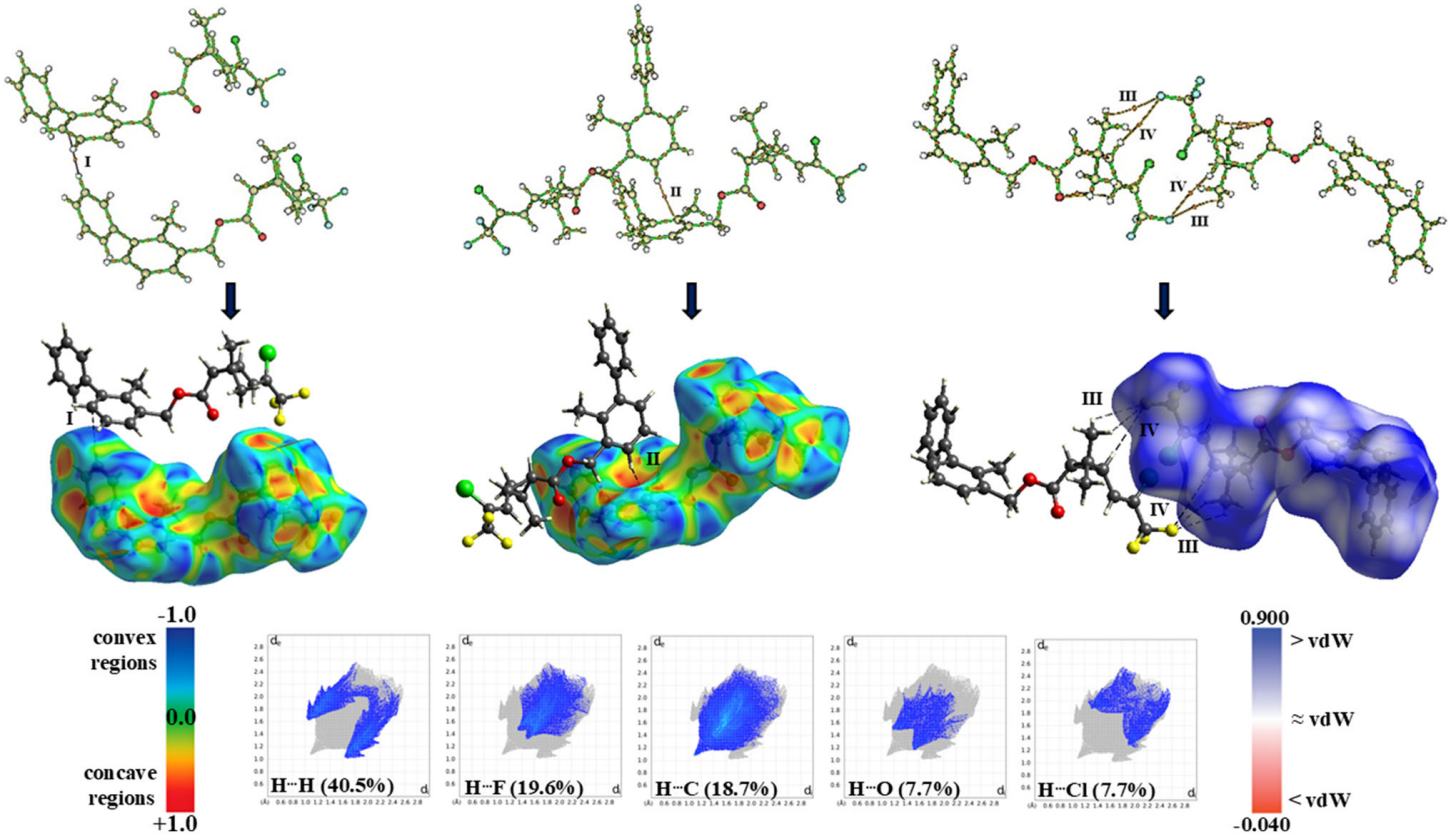
HS $d_{\text{norm}}$ highlighting possible intermolecular contacts, and the quantification of these interactions through fingerprint plots. **Interactions: (I)** C_3_–H_3_…Cg2; (**II**) C_10_–H_10_…Cg2; (**III**) C_20_–H_20B_…F_1_ and (**IV**) C_17_–H_17_…F_1_.

The crystal packing is stabilized exclusively by weak intermolecular interactions, namely C–H···π and C–H···F interactions (Figure S1). A prominent C–H···π interaction, C_10_–H_10_···Cg2, is characterized by an H···Cg distance of 2.613 Å, a C···Cg distance of 3.562 Å, and a nearly linear C–H···Cg angle of 177.67°, indicating a directional and structurally significant π‐accepting interaction. Complementary QTAIM analysis revealed the presence of a bond path (BP) between C_10_–H_10_ and C_12_ (Cg2) (II) (Figure 4), confirming the existence of this interaction. The electron density in the internuclear region is very low (ρ < 0.1 a.u.), indicating electron depletion at the BCP, a feature characteristic of weak, noncovalent interactions. According to the findings by Nakanishi and colleagues [65, 66], such topological parameters are consistent with a van der Waals interaction (Table 2). Furthermore, values of the |v|/G ratio less than 1.0 suggest that this is a closed–shell interaction [67]. The NBO analysis indicates that the C_10_–H_10_···Cg2 interaction is weakly stabilized through a hyperconjugative interaction of the type π(C_11_–C_12_) → $\sigma^{*}$(C_10_–H_10_), with an associated stabilization energy of 0.09  kcal·mol^−1^. That the interaction arises primarily from overlap between the π system and the $\sigma^{*}$(C_10_–H_10_) orbital, contributing 40.83% and 32.68%, respectively.

**TABLE 2:** Topological parameters of the QTAIM of BF interactions obtained at the M06‐2X/6‐311++G(d,p) level of theory.

**Interaction**	ρ(r) a **(a.u.)**	$\nabla^{2}\rho$ b **(a.u.)**	G(r) c **(a.u.)**	v(r) d **(a.u.)**	H(r) e **(a.u.)**	$\frac{\left|v\right|}{G}$	**Interaction type**
(I) C_3_–H_3_…C_8_ (Cg2)	0.0059	0.0168	0.0035	−0.0029	0.0007	0.8	van der Waals
(II) C_10_–H_10_…C_12_ (Cg2)	0.0066	0.0192	0.0041	−0.0034	0.0007	0.8	van der Waals
(III) C_20_–H_20_…F_1_	0.0038	0.0179	0.0035	−0.0025	0.0010	0.7	van der Waals
(IV) C_17_–H_17_…F_1_	0.0047	0.0161	0.0032	−0.0023	0.0009	0.7	van der Waals

a total electron density*.*

b Laplacian of electron density.

c kinect energy of electron density.

d potential energy of electron density.

e total electronic energy density.

A second C–H···π interaction, C_3_–H_3_···Cg2 (H···Cg distance of 2.836 Å and C–H···Cg angle of 144.8°), is comparatively weaker than the primary π interaction but still contributes to the stabilization of the supramolecular arrangement. QTAIM analysis also indicated the formation of a BP between C_3_–H_3_ and C_8_ (Cg2) with ρ(r) similarly depleted at the BCP. In this case, the ρ(r) value at the BCP is lower than that observed for the C_10_–H_10_···Cg2 interaction, confirming its weaker noncovalent character, with van der Waals character. Nevertheless, this interaction is slightly more stabilized from an orbital interaction perspective, as evidenced by the NBO analysis, which reveals a hyperconjugation π(C_8_–C_9_) → $\sigma^{*}$(C_3_–H_3_) with a $E_{i\to j^{*}}^{\left(2\right)}$ of 0.12  kcal·mol^−1^. The $\sigma^{*}$(C_3_–H_3_) and π orbitals contribute 40.36% and 35.33%, respectively, to this interaction.

C–H···F interactions are also observed, with H···F distances ranging from ≈2.95–3.06 Å and interaction angles spanning from about 103° to 142°. QTAIM analysis revealed the presence of two bond paths supporting these contacts: one associated with the C_17_–H_17_···F_1_ interaction and another involving the C_20_–H_20_···F_1_ interaction, both linking the tricyclic moiety to the fluorinated fragment. However, the electron densities in the corresponding internuclear regions are significantly lower than those observed for the C–H···π interactions discussed above, indicating an even weaker interaction regime. Consistently, NBO analysis shows no significant donor–acceptor hyperconjugative interactions stabilizing the C–H···F contacts. The relatively long H···F separations combined with their limited angular directionality suggest that these interactions are very weak in nature, being predominantly governed by dispersive and long‐range electrostatic contributions rather than by directional orbital interactions.

The electron density analysis based on HS corroborated the intermolecular contacts previously identified through geometric parameters, indicating that the C_10_–H_10_···Cg2 interaction is the most significant. This contact exhibits a distance shorter than the sum of the van der Waals radii, as evidenced by the red regions observed on the $d_{\text{norm}}$ surface (Figure 4). The white‐colored regions correspond to weaker interactions, with contact distances close to van der Waals separations, such as the C–H···F contacts. The 2D fingerprint plots revealed that BF is predominantly stabilized by interactions involving hydrogen atoms, with H···H contacts accounting for ≈40.5%, followed by hydrogen–fluorine contacts (H···F ≈ 19.6%) and π‐system interactions of the C–H···π type (≈18.7%). Additional contributions arise from H···O (≈7.7%) and H···Cl (≈7.7%) contacts, resulting in a cumulative contribution of ≈94.2% of all intermolecular contacts in BF.

These results confirm that the solid‐state structure corresponds to the 1*R*,3*R*/1*S*,3*S* racemic pair, with no crystallographic resolution of all four stereoisomers. Although crystallization enforces racemic packing in the solid state, molecular‐level differences among stereoisomers remain highly relevant in biological and environmental contexts, where interactions typically occur in solutions or within heterogeneous matrices.

### 3.2 Molecular Modeling Description

**FIGURE 5:**
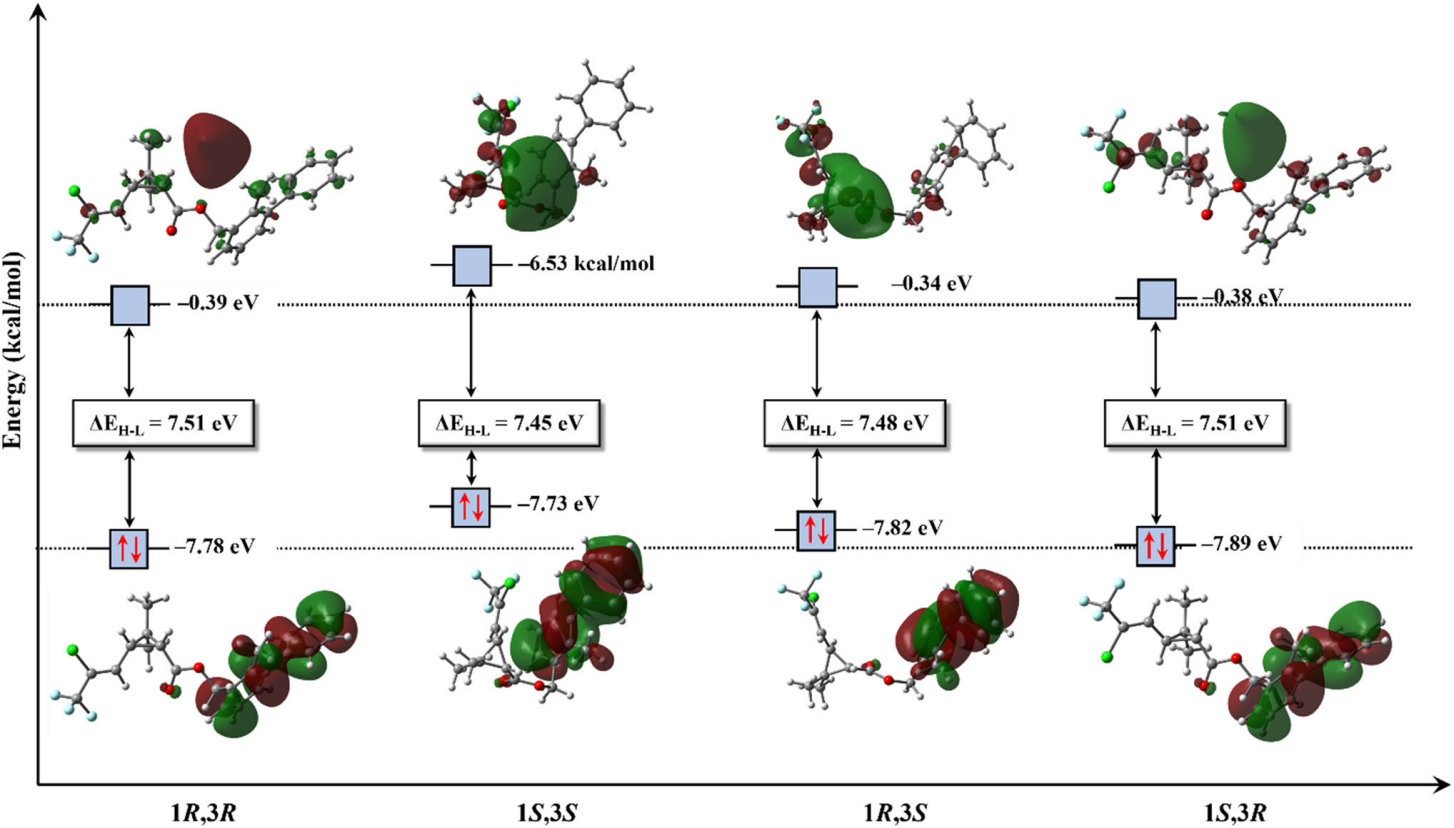
Isosurfaces of the HOMO and LUMO for the BF stereoisomers (isovalue = 0.02 a.u.). HOMO = Highest occupied molecular orbital; LUMO = lowest unoccupied molecular orbital.

The FMO isosurfaces of BF stereoisomers are shown in Figure 5, while the corresponding orbital energies are shown in Table 3. In all cases, HOMO exhibits predominantly π character, associated with the aromatic systems of the molecule. Analysis of the HOMO energies indicates that the 1*S*,3*S* stereoisomer possesses the least stabilized HOMO, implying a lower ionization energy and, consequently, a greater propensity for oxidation or electron‐donation processes. This interpretation is corroborated by the higher value of the chemical potential (μ) calculated for this stereoisomer, reinforcing its more electronically reactive character.

**TABLE 3:** Chemical reactivity descriptors calculated for the BF stereoisomers at the M06‐2X/6‐311++G(d,p) level of theory. All values are reported in eV.

Descriptor	1*R*,3*R*	1*S*,3*S*	1*R*,3*S*	1*S*,3*R*
HOMO energy, $E_{H}$	−7.891	−7.732	−7.823	−7.890
LUMO energy, $E_{L}$	−0.386	−0.283	−0.341	−0.383
Energy gap, $\Delta E_{H-L}$	7.505	7.449	7.481	7.507
Ionization energy, I	7.891	7.732	7.823	7.890
Electronic affinity, A	0.386	0.283	0.341	0.383
Electronegativity, χ	4.138	4.008	4.082	4.136
Chemical potential, μ	−4.138	−4.008	−4.082	−4.136
Chemical hardness, η	3.752	3.724	3.741	3.753
Electrophilicity index, ω	2.282	2.156	2.227	2.279

In contrast, LUMO displays a diffuse spatial distribution, characteristic of Rydberg‐type orbitals. Orbitals with this spatial profile are associated with relatively higher energies and reduced efficiency of direct orbital overlap with donor species, which tends to decrease the effectiveness of localized electron acceptance processes. Nevertheless, the presence of a more stabilized LUMO may favor long‐range electrostatic or dispersive interactions and influence electronic polarization effects [68, 69, 70]. In the case of BF, the 1*R*,3*R* stereoisomer exhibits the most stabilized LUMO, suggesting a slightly greater tendency to accommodate electronic polarization and for acting as an electron acceptor in intermolecular interactions.

The energy gap, $\Delta E_{H-L}$, defined as the difference between the LUMO ($E_{L}$) and HOMO ($E_{H}$) energies is a widely used global descriptor for assessing electronic stability and chemical reactivity. The results show that the 1*S*,3*R* stereoisomer presents the largest $\Delta E_{H-L}$, associated with a higher chemical hardness (η), characterizing a more electronically stable, less polarizable, and less perturbation‐sensitive structure. In contrast, the 1*S*,3*S* stereoisomer exhibits the smallest energy gap, lower chemical hardness, and higher polarizability, and is therefore the softest and most reactive stereoisomer in the set analyzed. Although these electronic differences are subtle, they may be highly relevant from biological and environmental perspectives.

According to the hard and soft acids and bases theory and conceptual DFT, softer and more polarizable molecules tend to interact more efficiently with enzyme active sites, biological receptors, and heterogeneous surfaces, potentially exhibiting higher affinity for molecular targets and greater susceptibility to metabolic and degradation processes. Conversely, harder and more electronically stable stereoisomers tend to display lower chemical reactivity and greater environmental persistence. Thus, the variations observed in the frontier orbitals and global reactivity descriptors of BF stereoisomers provide a consistent theoretical basis for rationalizing possible differences in chemical behavior, toxicity, and environmental fate [71, 72].

The global electrophilicity index (ω) indicates that all BF stereoisomers can be classified as strong electrophiles (ω > 1.5 eV), according to proposed by Domingo‐Pérez and colleagues [73, 74]. Among the stereoisomers analyzed, the 1*R*,3*R* isomer exhibits an electrophilic character ≈5.84% higher than that of the 1*S*,3*S* isomer. This difference can be attributed to the conformational arrangement of the 1*S*,3*S* stereoisomer in its ground state, which promotes a spatial redistribution of the electrostatic potential and reduces the exposure of regions with higher electron density. Similarly, the 1*S*,3*R* isomer displays a ω≈ 2.33% lower than that of the 1*R*,3*S* isomer, indicating that subtle conformational variations directly influence global reactivity descriptors.

**FIGURE 6:**
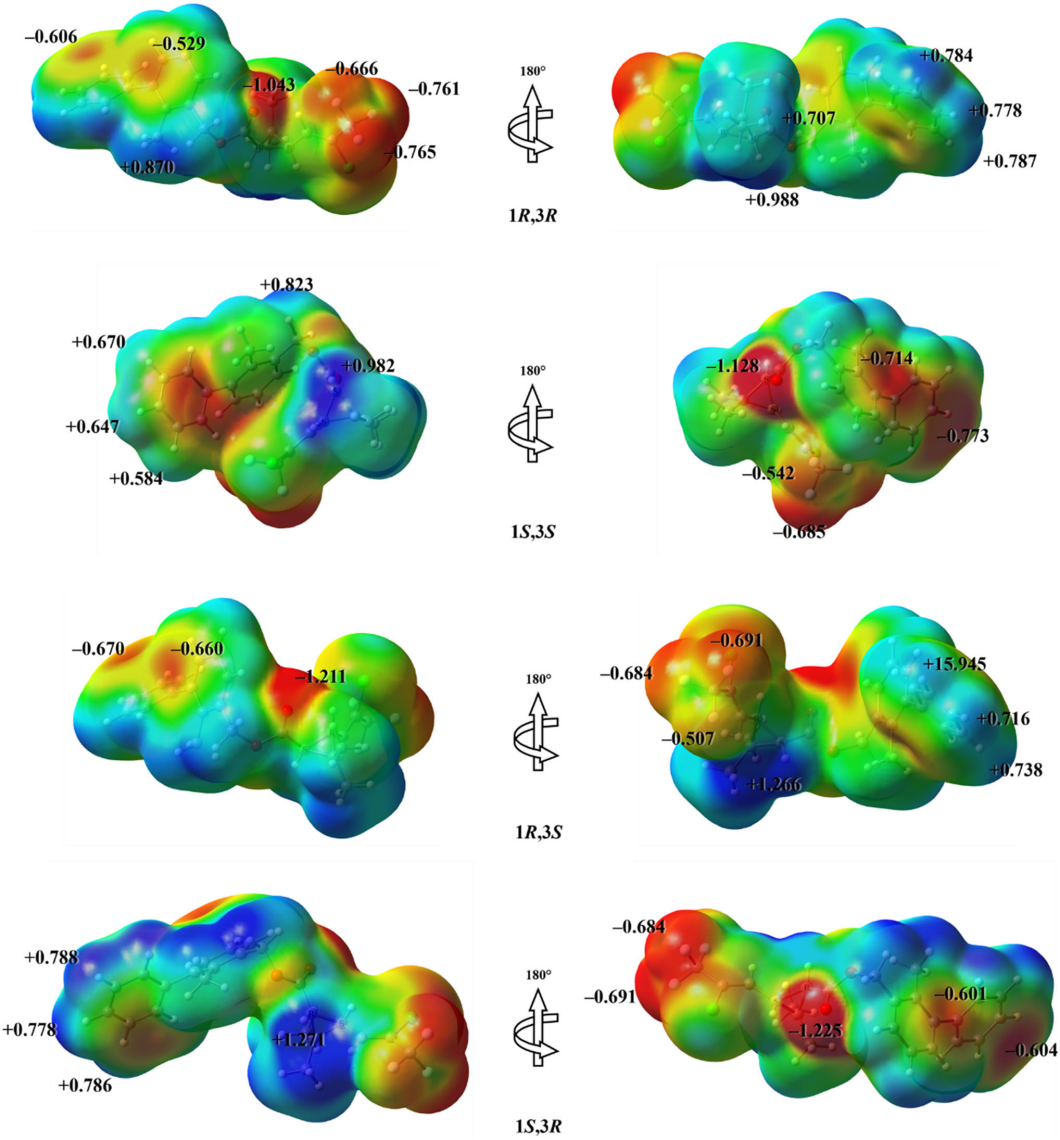
MEP surfaces mapped onto the electron density isosurface at ρ(r) = 4.0 × 10^−4^ electron/Bohr^3^ contour of the total SCF electronic density for the BF stereoisomers, obtained at the M06‐2X/6‐311++G(d,p) level of theory. MEP = Molecular electrostatic potential; SCF = self‐consistent field.

The MEP maps, shown in Figure 6, support this analysis by highlighting the preferentially nucleophilic (red) and electrophilic (dark blue) regions of the stereoisomers, as well as the extreme V(r) values associated with these regions. For all stereoisomers, the electrostatic potential is relatively evenly distributed over the molecular surface, with V(r) values close to neutrality, indicating an overall apolar character. This observation is quantitatively confirmed by the molecular polarity index (MPI), which reveals that the BF stereoisomers exhibit polarity comparable to that of benzene, a molecule classically regarded as apolar (Figure S2). Among the isomers examined, 1*R*,3*R* presents the highest MPI value, ≈9.89% higher than that of benzene, whereas the 1*S*,3*S* isomer shows the closest value, being only 4.59% higher. This trend is also reflected in the surface area analysis, which shows that the fraction of nonpolar surface area of the stereoisomers is similar to that of benzene, with only modest reductions: ≈2.29% for the 1*S*,3*S* isomer and 9.16% for the 1*R*,3*R* isomer. These results reinforce the predominantly hydrophobic character of BF, which is consistent with its high environmental persistence.

The MEP maps further allow the identification of sites of enhanced local reactivity. The most nucleophilic regions are mainly associated with the O atom of the carbonyl group and are most pronounced in the 1*S*,3*R* stereoisomer, for which the minimum V(r) value reaches −1.225 eV. In contrast, the most electrophilic site of this same stereoisomer is located at the hydrogen atom bonded to C16, with a maximum V(r) value of + 1.271 eV, indicating a region highly susceptible to electrostatic interactions with nucleophilic species. Complementary, condensed Fukui indices were employed to quantify local reactivity toward nucleophilic $\left(f^{+}\right)$, electrophilic $\left(f^{-}\right)$, and radical $\left(f^{0}\right)$ attacks (Figure S3). Overall, the local reactivity profiles exhibit highly consistent patterns among the stereoisomers, confirming that the global electronic distribution of the molecule is dominated by the aromatic system and strongly electronegative groups, such as halogen substituents. Nevertheless, subtle differences related to stereochemistry and molecular conformation modulate the magnitude of the indices at specific atomic sites, reflecting local redistributions of electron density.

The sites most susceptible to nucleophilic attack are predominantly located at the chlorine atom and at atoms C_21_ and C_22_, as well as at the aromatic carbons C_3_, C_4_, and C_5_. This behavior can be rationalized by the strong inductive effect of the halogen substituents, which polarize adjacent bonds and enhance the ability of these regions to accommodate additional electron density. In the 1*R*,3*S* and 1*S*,3*R* stereoisomers, the $f^{+}$values at C_21_ and C_22_ are slightly higher, suggesting that conformational rearrangements favor greater electronic exposure of these sites, making them more accessible to nucleophilic attack.

In contrast, the preferred sites for electrophilic attack are systematically observed at the aromatic carbons, particularly C_12_ and C_7_, followed by C_8_, C_9_, and C_10_. These atoms are part of the conjugated π system, in which electron density can be removed or redistributed with a lower energetic penalty. The high magnitude of $f^{-}$at these sites reflects the ease with which the aromatic system acts as an electron donor, in agreement with the π character of the HOMO discussed previously. Finally, the radical attack indices display an intermediate behavior, closely following the sites highlighted by $f^{-}$. The highest $f^{0}$ values are again concentrated at the aromatic carbons C_12_, C_7_, C_8_, C_9_, and C_10_, which is consistent with the ability of the π system to stabilize unpaired electron density through resonance. Consequently, these atoms emerge as preferential targets in radical‐mediated processes, such as oxidative degradation or photodegradation reactions, which are relevant to the environmental fate of BF.

As the relevance of chiral pesticides has become increasingly recognized, numerous experimental studies have focused on their enantioselective behavior across environmental and biological processes, with toxicology playing a central role in risk assessment. In this context, BF has been widely reported to exhibit pronounced enantioselective toxicity [5, 6, 21, 24]. Liu and Li reported that cytotoxicity and apoptosis induction in HepG2 cells are primarily attributed to the 1*S*,3*S* enantiomer [75]. Using a nontargeted metabolomics approach, Liao and collaborators [6] investigated the molecular mechanism of the differential toxic effects of BF enantiomers in human L02 liver cells, revealing distinct metabolic responses, with 51 metabolites altered by 1*S*,3*S* and 36 by 1*R*,3*R*.

Beyond hepatic toxicity, BF enantiomers also differentially affect endocrine‐related pathways. In rat ovarian cells, 1*S*,3*S* significantly reduced progesterone and PGE_2_ secretion in granulosa cells via the PKC signaling pathway. Molecular docking analyses indicated that although both enantiomers bind to the ATP‐binding pocket of PKC, only the 1*S*,3*S* enantiomer forms a stabilizing hydrogen bond with Lys368, providing a mechanistic rationale for its enhanced biological activity [20]. Consistently, in human breast carcinoma cells, exposure to the 1*S*,3*S* enantiomer resulted in a more pronounced upregulation of the estrogen‐responsive gene PS2 compared with the 1*R*,3*R* enantiomer, indicating a higher estrogenic activity of the 1*S*,3*S* isomer [76]. This trend was further corroborated, by enhanced proliferative responses by 1 in breast carcinoma cells and increased vitellogenin production in *J. medaka*, suggesting mediation through classical estrogen receptor‐dependent pathways [77].

In contrast, toxicity toward aquatic organisms is predominantly driven by the 1*R*,3*R* enantiomer. Pyrethroids are known to be substantially more toxic to fish than to mammals and birds, and within this framework, BF displays marked enantioselective toxicity in aquatic species. Acute toxicity assays indicate that lethality in *Ceriodaphnia dubia* is largely driven by the 1*R*,3*R* enantiomer, which exhibits ≈40‐fold greater toxicity than the corresponding 1*S*,3*S* form [78]. Similarly, in *Daphnia magna*, the lowest observed effect concentrations for survival and fecundity were ≈40‐ and 80‐fold lower for 1*R*,3*R* than for 1*S*,3*R* after 7 and 14 days of exposure, respectively. In line with these observations, subsequent studies demonstrated that the accumulation of 1*R*,3*R* in *D. magna* was ≈14–40 times higher than that of the 1*S*,3*R* enantiomer, indicating that enantioselective uptake and bioaccumulation play a key role in driving chronic toxicity in aquatic organisms [20, 22].

The experimental observations are naturally accounted for by the theoretical analysis developed herein, pronounced stereoelectronic differentiation among the BF stereoisomers. In particular, the 1*S*,3*S* enantiomer presents a higher‐energy HOMO, resulting in a reduced ionization energy and an enhanced ability to participate in electron‐donation and oxidative processes. This feature, combined with its smaller HOMO–LUMO energy gap, lower chemical hardness, and increased polarizability, characterizes the 1*S*,3*S* isomer as the softest and most electronically responsive species within the stereoisomeric set.

In addition to enantioselective differences in bioaccumulation and endocrine activity, extensive experimental evidence indicates that BF induces oxidative stress through excessive reactive oxygen species (ROS) generation, with pronounced enantioselectivity favoring the 1*S*,3*S* stereoisomer. In this context, oxidative damage emerges as a relevant mechanism of toxicity. Both in vitro and in vivo studies show that exposure to 1*S*,3*S* leads to stronger modulation of antioxidant defense systems and activation of apoptosis‐related pathways, consistent with the enhanced softness and polarizability identified for this enantiomer. In contrast, these properties are consistent with a lower tendency to directly induce oxidative damage, while favoring environmental persistence and bioaccumulation‐driven toxicity in aquatic systems. The DFT results show that this stereoelectronic differentiation provides a molecular‐level interpretation of the divergence between oxidative stress‐driven toxicity associated with 1*S*,3*S* and persistence‐driven toxicity governed by 1*R*,3*R*.

### 3.3 Regulatory Considerations

The combined findings from the solid‐state description and molecular modeling analyses suggest that the stereochemistry of BF may influence its toxicity, environmental fate, and biological performance. The comparative analysis of national and international regulations shows that BF regulation still does not address stereospecific aspects, treating the active substance as a single entity despite evidence that its enantiomers exhibit significant differences in toxicity, environmental persistence, and reactive behavior. In Brazil, pesticide regulation is governed by Law 14,785/2023 and Decree 4,074/2002, with responsibilities shared among the Ministry of Agriculture, Livestock and Food Supply (MAPA), the Brazilian Health Regulatory Agency (ANVISA), and the IBAMA. BF is registered as a racemic mixture, and current legislation does not require stereospecific data or polymorph characterization, focusing instead on overall toxicological and ecotoxicological profiles [15, 16].

In the United States, the EPA regulates BF under FIFRA and completed an Interim Registration Review in 2020 [2, 4]. This review imposed mitigation measures such as buffer zones, spray drift reduction, and label changes to minimize human and ecological risks. However, the assessment treats BF as a single active ingredient, without differentiating enantiomers or requiring enantioselective toxicity data. In the European Union, BF was initially approved under Implementing Regulation (EU) 582/2012 but was later withdrawn from the market due to high aquatic toxicity and bioaccumulation concerns, as formalized in Regulation (EU) 2022/643 [19]. Despite its strict stance on ecotoxicological risk, EU regulations also do not address stereochemistry or polymorphism in pesticide risk assessment [4, 19, 79].

Although BF remains widely applied in agricultural contexts, uncertainties persist regarding its toxicity to nontarget organisms and potential cumulative environmental effects. The authors emphasize that current regulatory assessments often treat BF as a single entity, disregarding differences among stereoisomers or polymorphs, which may lead to underestimated risks. This evidence reinforces the need for more robust, stereospecific policies capable of accurately reflecting the toxicological and environmental impacts of the molecule. Aligning scientific findings with regulatory practices is therefore essential to advance toward more sustainable risk assessments and the development of safer pesticide formulations [80]. At the international level, FAO/WHO specifications and OECD guidelines provide harmonized frameworks for pesticide quality and risk assessment, but do not include explicit requirements for enantioselective evaluation or polymorph control, even though these factors can significantly influence environmental fate and toxicity [81]. Across all jurisdictions, the absence of stereospecific and polymorphic considerations in regulatory frameworks contrasts with growing scientific evidence on enantioselective toxicity and environmental persistence. This gap is critical because the commercialization of racemic mixtures may amplify unforeseen adverse impacts.

Overall, our results demonstrate that the four stereoisomers of BF possess distinctly different stereoelectronic profiles. In parallel, the computational descriptors clearly differentiate the 1*S*,3*S* stereoisomer, whose highly anisotropic electrostatic distribution, markedly reduced nucleophilicity, and distinct reactive sites align with toxicological reports identifying 1*S*,3*S* as the most toxic configuration to humans [6, 7]. Despite this clear stereochemical influence, current regulatory frameworks still treat BF as a single active ingredient. The computational evidence presented here shows that such an approach overlooks meaningful stereoelectronic and reactivity differences that can affect biological activity, toxicity, and environmental fate. These findings highlight the need for stereochemistry‐aware evaluation and regulation of pyrethroids, recognizing configuration as a primary determinant of molecular behavior.

## 4 Conclusion

This study provided a comprehensive characterization of BF, combining crystallographic analyses and theoretical modeling to elucidate structural features and stereoisomer‐dependent behavior. The results revealed that, although the enantiomers share identical intrinsic electronic properties in the isolated state, differences arise from the specific conformations adopted in the solid state. Variations in electrostatic potential distribution and nucleophilicity indices are associated with conformational constraints and intermolecular interactions imposed by crystal packing, especially for the 1*S*,3*S* isomer. These findings provide a molecular‐level framework to rationalize reported variations in biological activity, environmental persistence, and reactivity among BF stereoisomers, particularly in environments where conformational preferences and local interactions impose structural constraints analogous to those observed in the solid state. In this context, the results emphasize the role of environment‐dependent organization rather than intrinsic electronic differences. Additionally, the crystalline phase is stabilized as a racemic assembly of the 1*R*,3*R* and 1*S*,3*S* enantiomers, consistent with the symmetry requirements of the C2/c space group. However, current regulatory assessments, both in Brazil and other jurisdictions, do not consider the distinction between enantiomers or polymorphs, treating the active substance as a single entity. This regulatory gap, combined with the scientific evidence presented, highlights the need for policies that incorporate stereospecific criteria and guidelines for polymorph characterization, enabling a more accurate and sustainability‐oriented risk assessment. Overall, by integrating solid‐state structural analysis with theoretical electronic structure calculations, this work advances the understanding of BF stereoisomers and provides a consistent scientific basis for interpreting their environmental behavior, biological effects, and regulatory implications.

## Supporting Information

Additional supporting information can be found online in the Supporting Information section.

## Conflict of Interest

The authors declare no conflicts of interest.

## Supporting information

Supplementary Material
