# Supplementary material for: Insights Into Bifenthrin Stereoisomers and Their Regulatory Implications
Source: ChemistryOpen. 2026 Apr 20;15(5):e70212. doi: 10.1002/open.70212 (PMC13096588; doi:10.1002/open.70212)
Supplement: Supplementary file 1 — Supplementary Material [file OPEN-15-e70212-s001.pdf]

Insights into Bifenthrin Stereoisomers and their Regulatory Implications

Nayara C.M. Santos[1,2], Nayara D. Coutinho[1], Vitor S. Duarte[2], Anderson Catao[2], Antônio S.N. Aguiar[2,3], Lucas D. Dias[3], James O. Fajemiroye[3,4] & Hamilton B. Napolitano[2*]

[1]Laboratório de Polimorfismo Molecular, Faculdade SENAI Roberto Mange, Anápolis, Goiás, Brazil
[2]Theoretical and Structural Chemistry Group, State University of Goiás, Anápolis, GO, Brazil.
[3]Laboratório de Novos Materiais, Universidade Evangélica de Goiás, Anápolis, GO, Brazil.
[4]Institute of Biological Sciences, Federal University of Goiás, Goiânia, GO, Brazil

Corresponding authors: nayarachristyan@gmail.com, toninho.quimica@gmail.com, hbnapolitano@gmail.com,

Supplementary Information

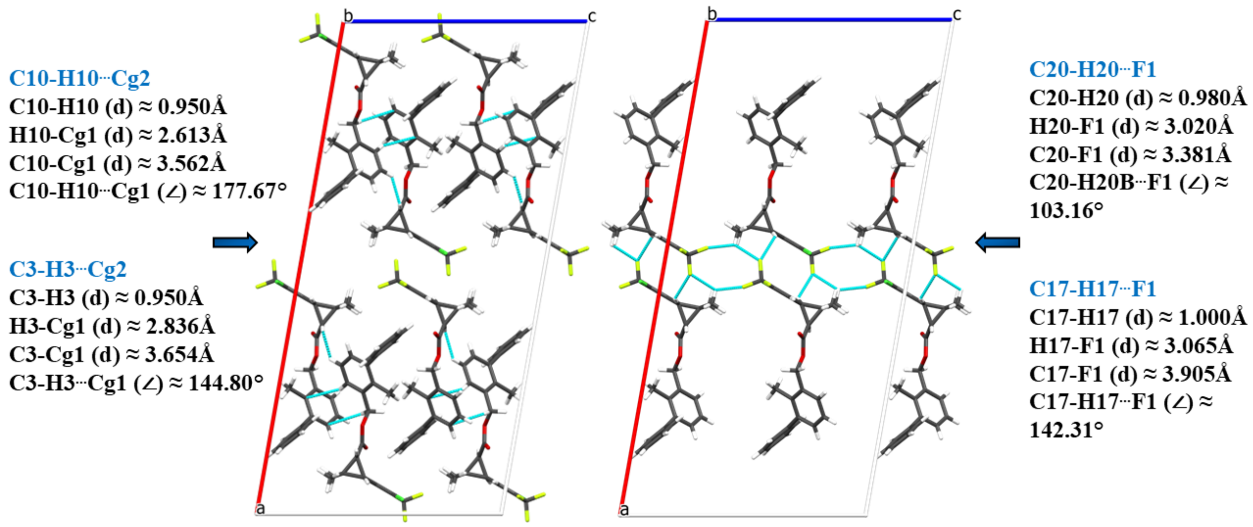

Figure S1. Supramolecular arrangement of bifenthrin in the unit cell, highlighting some intermolecular interactions.

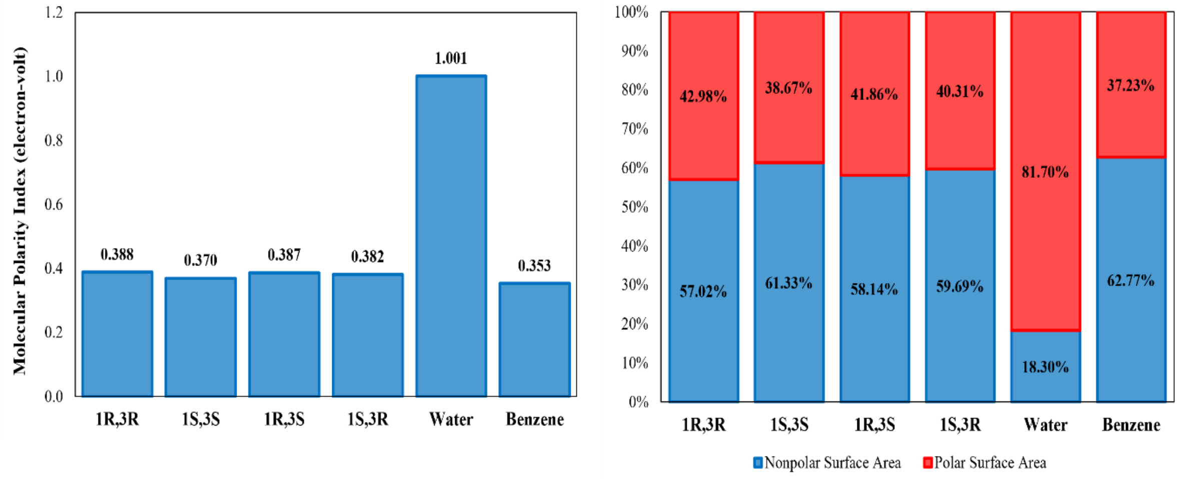

(a) **(b)**

Figure S2. Polarity analysis of bifenthrin stereoisomers, showing (**a**) the molecular polarity index (MPI) and (**b**) the distribution of polar and nonpolar surface areas. These results reinforce the hydrophobic nature of bifenthrin, given the dominant nonpolar character of its stereoisomers.

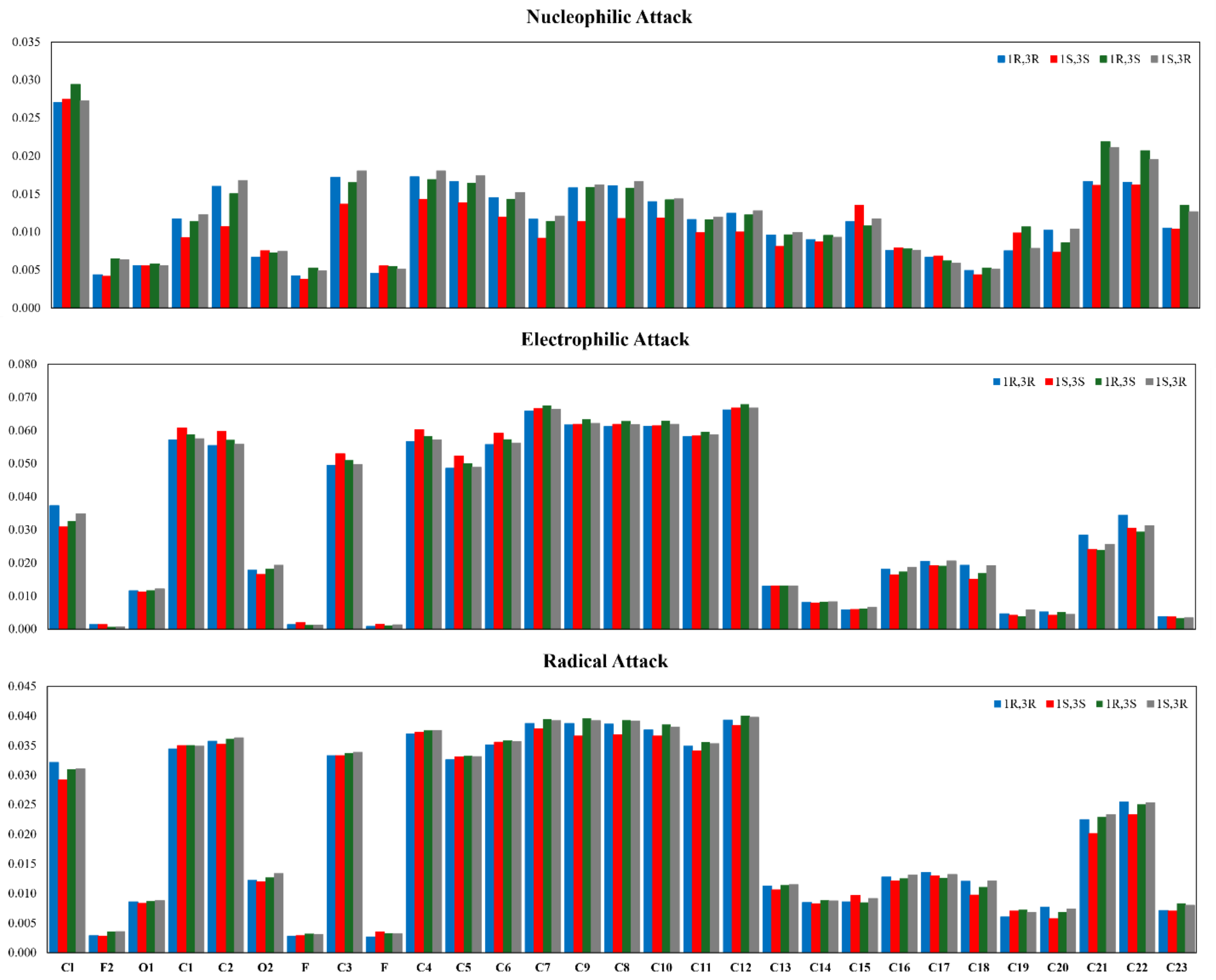

Figure S3. Condensed Fukui indices for the bifenthrin stereoisomers (1*R*,3*R*; 1*S*,3*S*; 1*R*,3*S*; and 1*S*,3*R*) calculated at the M06-2X/6-311++G(d,p) level of theory. The bar plots represent the susceptibility of each atomic site to nucleophilic (f^+), electrophilic (f^-), and radical (f^0) attacks.
